# Design of a new electrochemical aptasensor based on screen printed carbon electrode modified with gold nanoparticles for the detection of fumonisin B1 in maize flour

**DOI:** 10.1186/s12951-022-01745-7

**Published:** 2023-01-02

**Authors:** Behnaz Naghshbandi, Mohsen Adabi, Kamran Pooshang Bagheri, Hamid Tavakolipour

**Affiliations:** 1 Department of Food Science and Technology, North Tehran Branch, Islamic Azad University, Tehran, Iran; 2 Department of Metallurgy and Materials Science, Roudehen Branch, Islamic Azad University, Roudehen, Iran; 3 Venom and Biotherapeutics Molecules Lab, Medical Biotechnology Department, Biotechnology Research Center, Pasteur Institute of Iran, Tehran, Iran; 4 Department of Food Science and Technology, Sabzevar Branch, Islamic Azad University, Sabzevar, Iran

**Keywords:** Aptasensor, Fumonisin B1, Maize flour, Screen printed carbon electrode, Gold nanoparticles

## Abstract

A new aptasensor for detecting fumonisin B1 (FB1) in the maize samples was developed based on DNA- aptamer recognition and electrochemical technique. A thiol-modified single-stranded DNA (ss-HSDNA) was immobilized on a screen printed carbon electrode (SPCE) electrodeposited by gold nanoparticles (AuNPs). The morphology and structure of SPCE and AuNPs/SPCE were evaluated via scanning electron microscopy (SEM) equipped with energy dispersive spectroscopy (EDS). The SEM results demonstrated that the SPCE had a flat sheet-like structure, and the AuNPs were homogeneously electrodeposited on the SPCE. Cyclic voltammetry (CV) experiments in the [Fe(CN)_6_]^− 3/− 4^ solution were conducted to investigate each step of electrode modification as well as aptasensor performance. Aptamer-FB1 interaction prevented the electron transfer permitting the determination of FB1 in the range of 0.5–500 ng/mL with a low detection limit (0.14 ng/mL). The designed aptasensor was also shown high selectivity, acceptable repeatability and reproducibility, good long-term stability, and excellent recovery. Furthermore, there was a strong correlation between the findings achieved via the designed aptasensor and high performance liquid chromatography (HPLC). Therefore, a simple construction process and satisfactory electrochemical performance of the proposed aptasensor have a great potential for the detection of FB1 in maize samples.

## Introduction

Mycotoxins, mainly produced by the secondary metabolism of three fungal genera, namely Aspergillus, Penicillium, and Fusarium, are toxic compounds that may be found in food and feed [1]. Therefore, the possibility of the presence of mycotoxin contamination in various foodstuffs is a threat to both humans and animals that can cause disease and even death [2]. Among the prevalent mycotoxins, fumonisin B1 (FB1) is the dangerous contaminant that can infect different agricultural products such as rice, maize, peanut and wheat [3]. According to the International Agency for Research on Cancer (IARC), FB1 has been classified in group 2B as possibly carcinogenic to humans [4]. It has been reported that the FB1 driven by the consummation of moldy cereals can result in human esophageal and liver cancers. Hence, the maximum residue limits (MRLs) of FB in foodstuffs have been set by many countries. For instance, US Food and Drug Administration (FDA) has established the MRLs of 2000 µg/kg for type B-fumonisin in agricultural products, whereas European Union legislation has set the MRLs as 4000 µg/kg for fumonisin B in cereal [5–7]. Thus, it seems necessary to detect FB1 in food or agricultural products.

Up to now, many studies have been conducted to detect FB1 in food or feed. In fact, there are useful analytical approaches such as thin layer chromatography (TLC), enzyme-linked immunosorbent assay (ELISA), liquid chromatography-mass spectrometer (LC–MS), and high performance liquid chromatography (HPLC) for FB1 detection in agricultural crops [8–11]. Although these methods have good advantages like high sensitivity and specificity, they are time-consuming and need costly instruments as well as experienced technicians. Because of these shortcomings, different alternative methods have been developed [12]. In recent decades, the biosensor, due to its unique advantages such as low cost, portability, rapid response, and simple operation, has been widely used in mycotoxins detection. Among various biosensors, the electrochemical biosensor has become an attractive tool for mycotoxin assay because of its high sensitivity, simple equipment, and easy miniaturization [13, 14].

Recent years have witnessed a considerable increase in the demand for the utilization of aptamer as a molecular recognition element in biosensors. Aptamers produced via a process known as the systematic evolution of ligands by exponential enrichment (SELEX) are short, single-stranded oligonucleotides that can bind to various target molecules through the formation of three-dimensional structures [15]. To date, several aptasenors have successfully been developed according to the FB1 aptamer designed by McKeague et al. [16]. For instance, Yue et al. developed aptamer-based suspension array for simultaneous recognition of FB1 and ochratoxin A (OTA) [17].

Despite many attempts to introduce a convenient and straightforward aptamer-based biosensor for monitoring FB1, there are still challenges to obtaining high sensitivity for FB1 detection. In order to improve the performance of aptamer-based biosensor, the combination of aptamer with nanostructured materials has been developed. Many studies have focused on gold nanoparticles (AuNPs) as a platform for the development of aptasensors due to their unique properties such as easiness in functionalization procedure, biocompatibility, chemical stability, high specific surface area and low toxicity [18–20]. The gold nanoparticles can be assembled onto different substrates such as gold, platinum, glassy carbon, and screen-printed carbon electrodes. Among them, screen-printed carbon electrode (SPCE) has received great attention for electrochemical detection because of their cost-effectiveness, portability, simplicity of mass production, and convenient pretreatment of the electrode [21]. To the best of authors’ knowledge, it is first time that the SPCE electrodeposited with AuNPs was used as a platform to immobilize the aptamer for detection of FB1 in maize flour. Therefore, using ss-HSDNA/AuNPs/SPCE to develop the high performance aptamer based electrochemical biosensor is an exciting challenge.

This research aimed to design a new electrochemical aptasensor for detecting FB1 in maize flour. For this purpose, the electrode was prepared using the immobilization of ss-HSDNA onto AuNPs electrodeposited on SPCE. The preparation steps of the electrode were investigated using the cyclic voltammetry technique. Furthermore, the detection of FB1 in maize flour samples was performed by means of the cyclic voltammetry method and HPLC.

## Experimental

### Materials and reagents

Screen-printed carbon electrodes were bought from DropSens (Spain). The screen-printed electrodes utilized in this research consisted typically of three electrodes: the carbon working electrode (WE), the carbon counter electrode, and the Ag/AgCl reference electrode (RE). Sulfuric acid (H_2_SO_4_), potassium chloride (KCl), magnesium chloride (MgCl_2_), calcium chloride (CaCl_2_), sodium chloride (NaCl), Tris–HCl, Hydrogen tetracholoroaurate (HAuCl_4_), methanol (CH_3_OH), sodium phosphate dibasic (Na_2_HPO_4_), potassium phosphate monobasic (KH_2_PO_4_), potassium ferrocyanide (K_4_[Fe(CN)_6_]), potassium ferricyanide (K_3_[Fe(CN)_6_]), 2-mercaptoethanol (2-MCE), zearalenone (ZEN), Aflatoxin M1 (AFM1) and Aflatoxin B1 (AFB1) were purchased from Sigma-Aldrich. FB1-aptamer ss-HSDNA sequence (5′ SH(CH_2_)_6_AGCAGCACAGAGGTCAGATGCGATCTGGATATTATTTTTGATACCCCTTT GGGAGACATCCTATGCGTGCTACCGTGAA-3′) was bought from Thermo Fisher Scientific. Ultrapure water was used to prepare all solutions.

### Preparation process of aptasensor

**Fig. 1:**
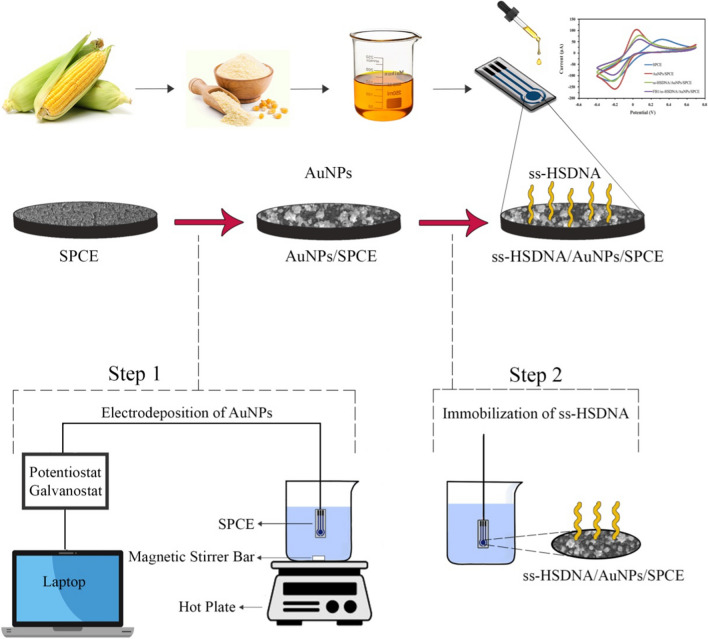
Schematic illustration of the preparation and performance of the electrochemical aptasensor

A schematic of the preparation and performance of the aptasensor was illustrated in Fig. 1. As shown in this figure, the process for the preparation of the ss-HSDNA/AuNPs/SPCE consists of two major steps described in the following sections:

#### Electrodeposition of gold nanoparticles on SPCE

The gold nanoparticles were applied onto the SPCE surface using the electrodeposition technique under a constant potential of − 0.4 V versus Ag/AgCl for 60 s in 5 mM HAuCl_4_ containing 0.1 M H_2_SO_4_ solution.

#### Immobilization of ss-HSDNA onto SPCE modified by AuNPs

The immobilization of ss-HSDNA on the AuNPs-modified electrode surface was carried out by immersion of AuNPs/SPCE in a solution containing 5 µM ss-HSDNA at the temperature of 36 ± 2 °C for 6 h. It should be noted that aptamer folding form was obtained by heating the aptamer at 94 °C for 5 min and followed by cooling with ice for 15 min. The prepared electrode was then washed with binding buffer (100 mM NaCl, 5 mM KCl, 2 mM MgCl_2_, 20 mM Tris–HCl and 1 mM CaCl_2_) to eliminate the unbounded aptamer. Afterward, 10 μL of 1 mM 2-MCE solutions were dropped on the modified electrode surface and incubated for 1 h to block the possible remaining active sites. Finally, the resulting electrode was stored at 4 °C until utilization.

### Working solution preparation

The working solutions of FB1, AFB1, AFM1, and ZEN at different concentrations were prepared by dissolving each of the mycotoxins powder in acetonitrile/H_2_O (50/50, v/v).

### Maize sample preparation

The maize flour was bought from local markets and analyzed using LC/MS to make sure that the maize flour was free from FB1. The maize flour was firstly ground to pass an 80-mesh sieve, followed by spiking with 50 µL drop of different working solutions of mycotoxins. The maize samples were then incubated for 24 h at room temperature. Afterward, the extraction of maize samples was carried out using 3 mL of methanol/water (20:80, v/v) by means of a horizontal shaker for 20 min at room temperature and centrifugal apparatus at 4 °C and 5000 rpm for 6 min. The supernatant was filtered through a 0.22 μm disposable syringe filter and then diluted with a binding buffer. The prepared maize samples were characterized by the electrochemical measurement explained in the “Measurement procedure” section.

### Characterization of the SPCE and AuNPs/SPCE

The surface morphology and chemical composition of the SPCE and AuNPs/SPCE were evaluated by means of a Vega Tescan scanning electron microscope (SEM) equipped with an energy dispersive X-ray spectrometer (EDS).

### Electrochemical measurements

All electrochemical measurements were conducted using μStat 400 potentiostat/galvanostat (DropSens, Spain) in a potential range of − 0.4–0.7 V at pH 7 and a scanning rate of 50 mV/s.

### Measurement procedure

To obtain the response of the aptasensor assay, ss-HSDNA/AuNPs/SPCE was put into the solution prepared according to Section “Maize sample preparation”. The aptasensor was dipped into 5.0 mM ferri/ferrocyanide ([Fe(CN)6]^3−/4−^) electrolyte containing 0.1 M KCl, and the CV measurements were carried out as described in the previous section.

## Results and discussion

### Characterization of microstructure of AuNPs/SPCE by SEM

**Fig. 2:**
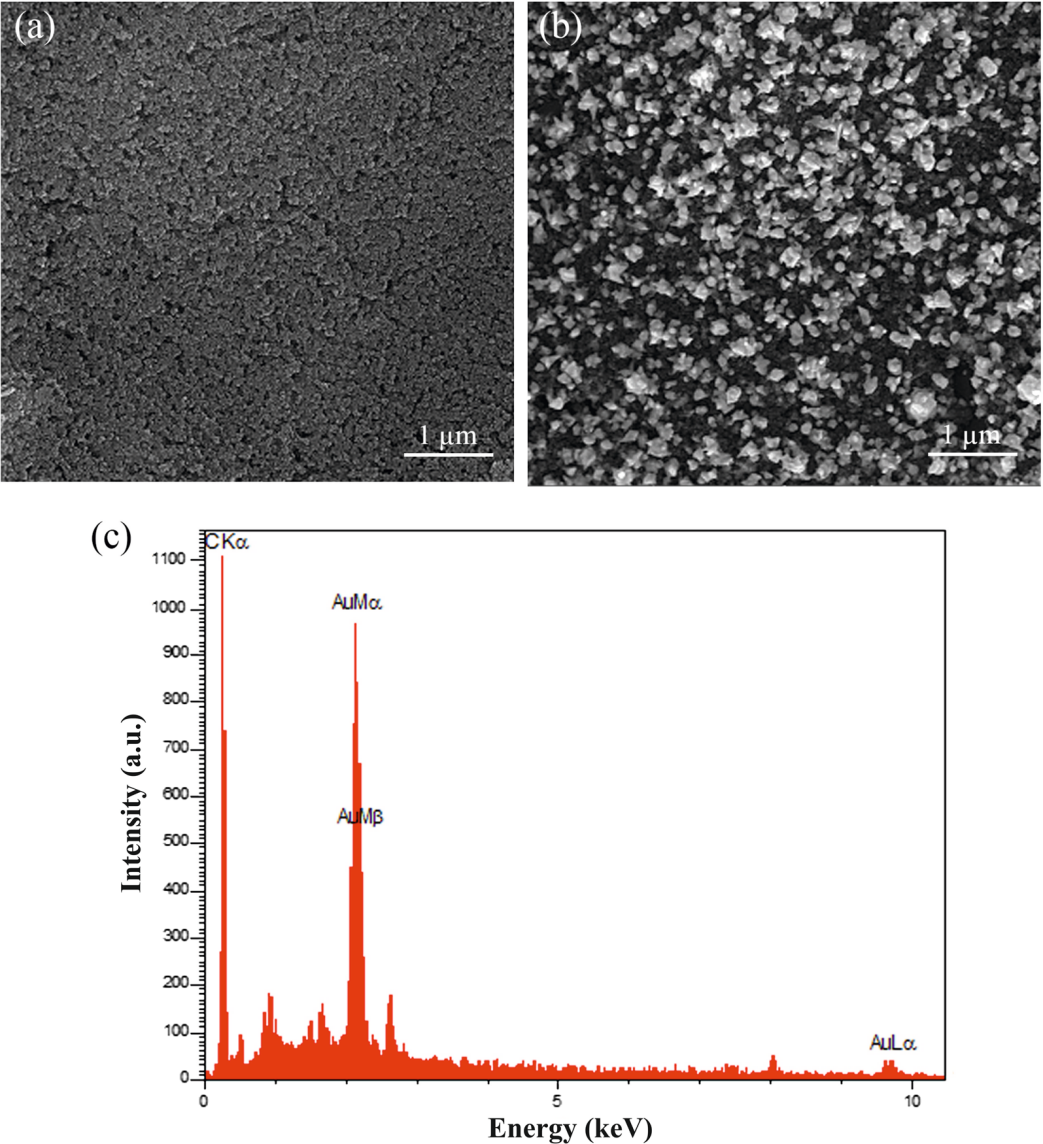
SEM images of **a** SPCE and **b** AuNPs/SPCE and **c** EDS analysis of the AuNPs/SPCE

The SEM images of SPCE and AuNPs/SPCE are shown in Fig. 2. As shown in this figure, the SPCE possessed a flat sheet-like structure, and the AuNPs homogeneously electrodeposited on the surface of SPCE were regular. Furthermore, the EDS analysis confirmed the deposition of gold nanoparticles.

### Electrochemical characterization of ss-HSDNA/AuNPs/SPCE aptasensor

**Fig. 3:**
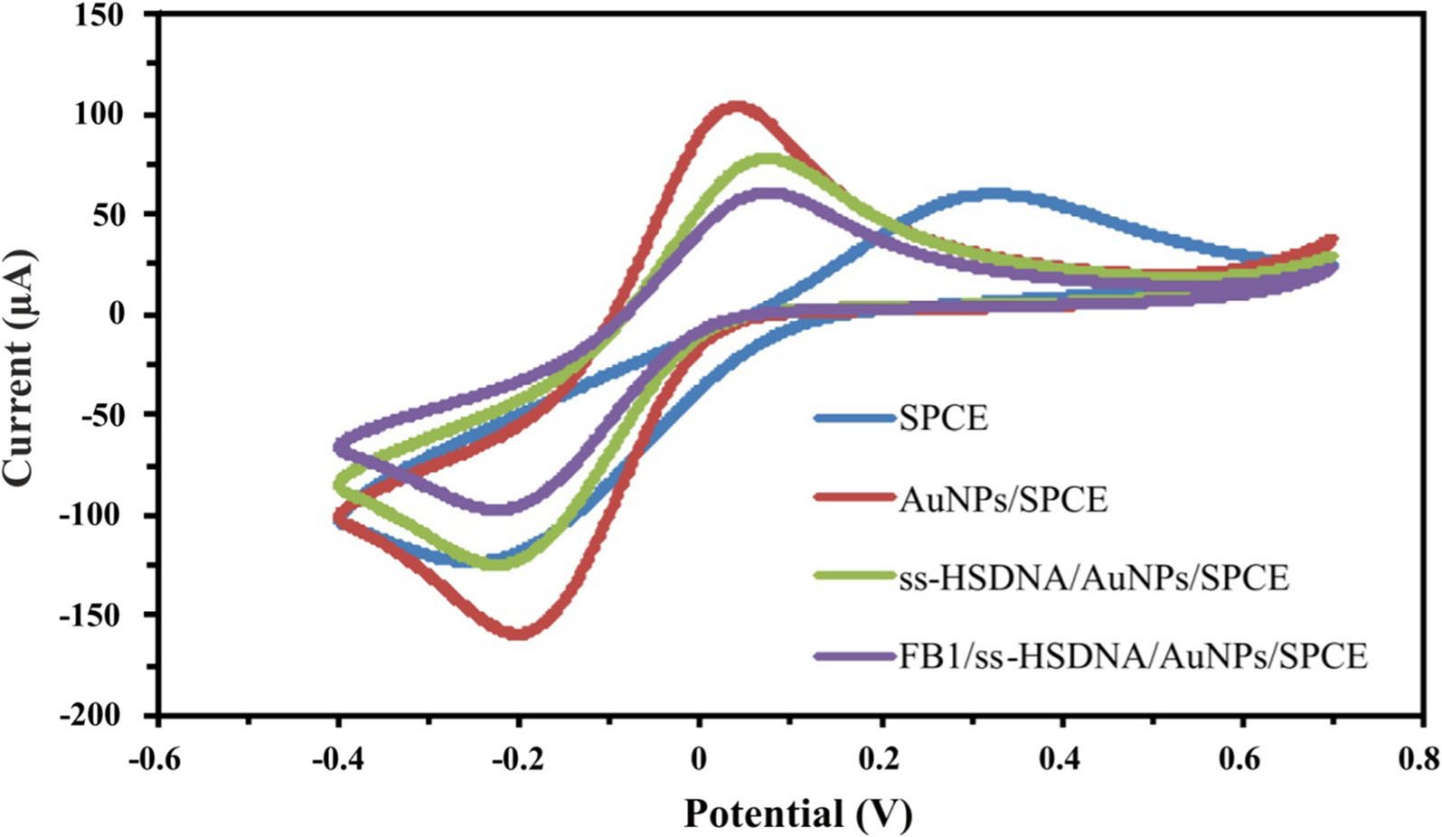
Cyclic voltammograms of the stepwise modified electrodes at pH 7 and a scanning rate of 50 mV/s

According to theory, the change in voltammetric peak current of CV curves occurs after each step of electrode modification due to the charge transfer resistance. The CV curves related to different electrodes, in the absence or existence of 50 ng/mL FB1, are displayed in Fig. 3. It can be clearly that the bare SPCE has a pair of well-defined redox peaks which is owing to oxidation/reduction of Fe[(CN)_6_]^− 3/− 4^. After the electrodeposition of AuNPs on the SPCE surface, an increase in the oxidation/reduction peaks is observed. This is due to the more electron transfer rate provided by a large surface area of gold nanoparticles. However, the voltammetric response of Fe[(CN)_6_]^− 3/− 4^ oxidation/reduction decreased as the ss-HSDNA was immobilized onto AuNPs/SPCE. This indicates that electron transfer between the surface of AuNPs/SPCE and Fe[(CN)_6_]^− 3/− 4^ solution was reduced due to the immobilization of ss-HSDNA. It is worth pointing out that the peak current was further decreased in the presence of FB1. It can be attributed to the blocking effect of FB1 on electron transfer to the electrode surface resulting from the formation of a target-aptamer complex. Hence, the change in resistance of electron transfer taking place during the Fe[(CN)_6_]^− 3/− 4^ oxidation/reduction on the interface of electrode/solution can be stated as a detection mechanism. This finding is in good agreement with the results reported by Chen et al. [22].

### Electrochemical aptasensor performance for FB1 detection

**Fig. 4:**
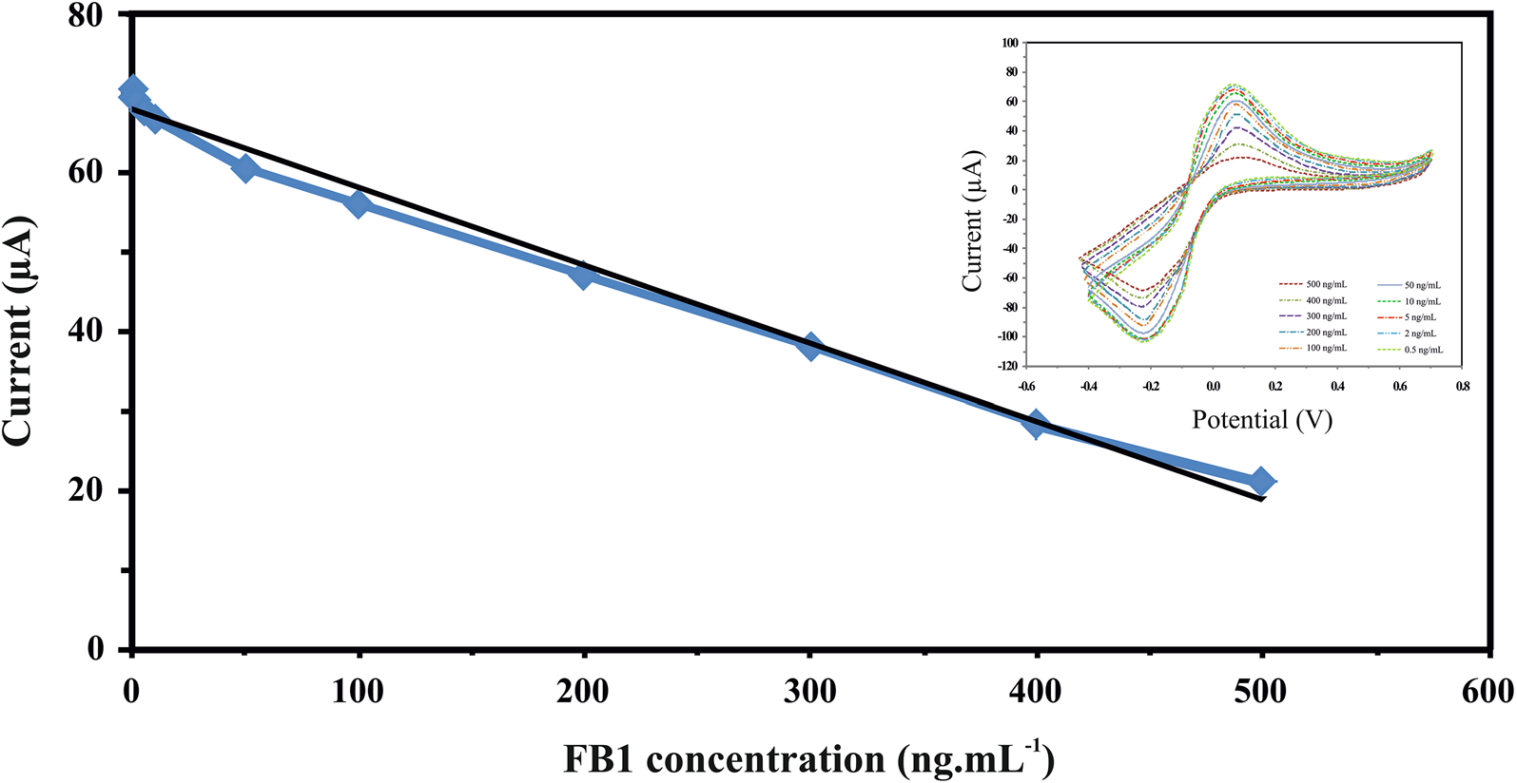
The dependence of peak current of the designed aptasensor on the FB1 concentration with a correlation coefficient of 0.991 (inset: Cyclic voltammograms of the aptasensor with FB1 concentration range from 0.5  to 500 ng/mL)

The measurement of peak current at different concentrations of FB1 was carried out by means of the cyclic voltammetric method. The peak current is plotted as a function of the average of three measurements for each FB1 concentration in Fig. 4. As seen in this figure, there is a good linear relationship between the peak current and the FB1 concentration in the range of 0.5–500 ng/mL. The limit of detection (LOD) and Limit of quantification (LOQ) calculated according to the following equation were 0.14 ng/mL and 0.46 ng/mL, respectively [23]:

1 $$\mathrm{LOD }=\frac{3\upsigma }{\left|\mathrm{s}\right|}$$

2 $$\mathrm{LOQ }=\frac{10\upsigma }{\left|\mathrm{s}\right|}$$

where σ represents the standard deviation of the blank’s response and s can be obtained from the slope of the regression line seen in Fig. 4. The linear working range and detection limit for FB1 achieved by different techniques were listed in Table 1. As shown in the table, the linearity range and detection limit obtained in this study are comparable to the results reported in other literature. This indicates that the CV aptasensor is a promising technique for the detection of FB1 in feeds and foods.

**Table 1:** Analytical parameters obtained at various aptasensors

Analytical method	LOD	Linear range	References
Fluorescence resonance energy transfer aptasensor	0.1 ng/mL	0.1–500 ng/mL	[24]
Aptamer-Photonic crystal encoded suspension array	0.16 pg/mL	0.001–1 ng/mL	[17]
Aptamer-based microcantilever array biosensor	0.33 ng/mL	0.1–40 µg/mL	[25]
Aptamer based colorimetric assay	0.3 ng/mL	0.5–300 ng/mL	[26]
Electrochemical Immunosensor	2 pg/mL	0.01–1000 ng/mL	[27]
Electrochemical aptasensor	3.4 pg/mL	0.01–50 ng/mL	[28]
Electrochemical aptasensor	10 pg/mL	10^–11^-10^–4^ g/mL	[29]
CV aptasensor	0.14 ng/mL	0.5–500 ng/mL	This work

### Selectivity of the designed aptasensor for FB1

**Fig. 5:**
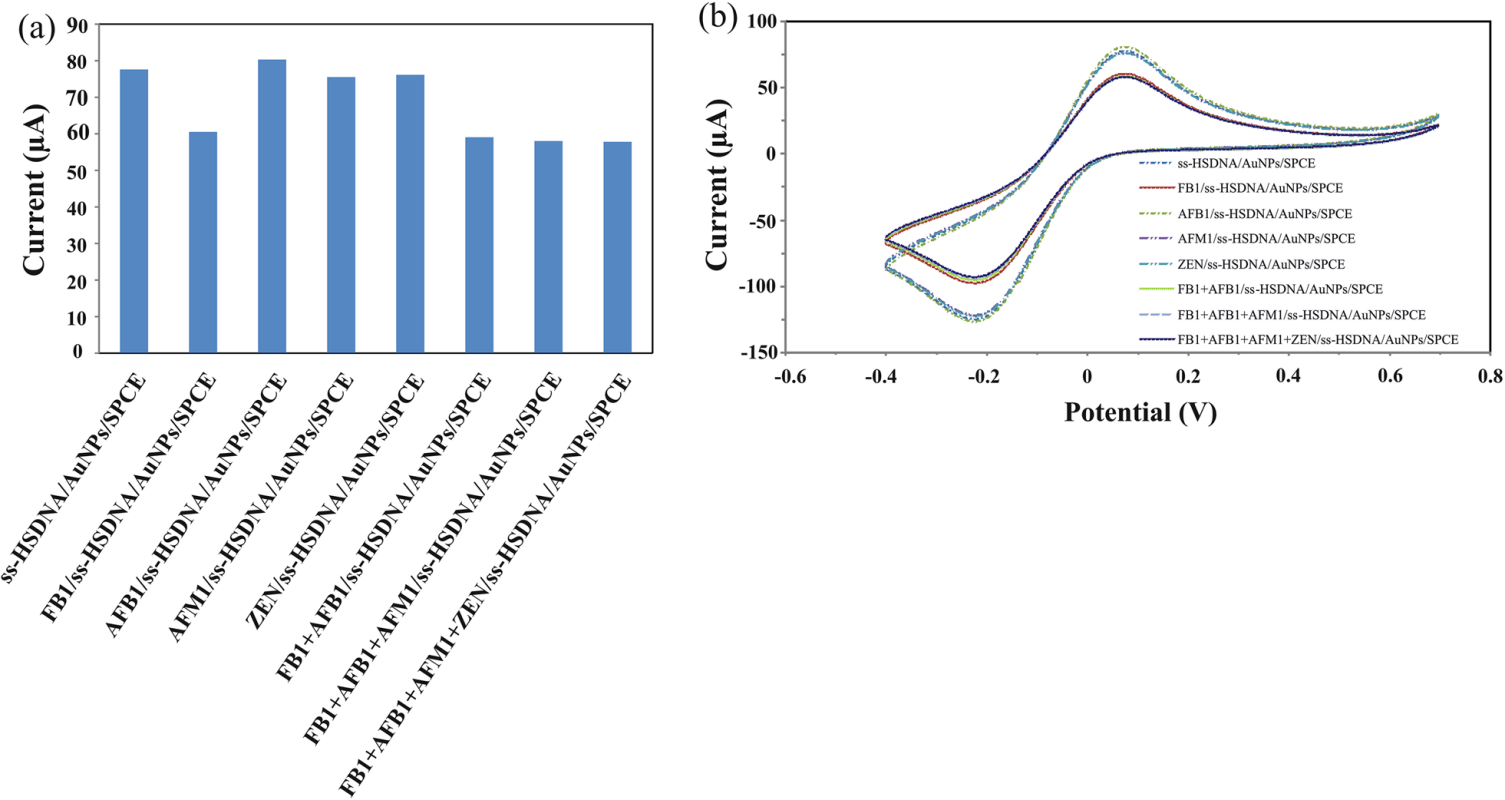
**a** Selectivity and **b** cyclic voltammograms of the designed aptasensor toward FB1 in presence of other mycotoxins (AFB1, AFM1 and ZEN) at the same concentration

Selectivity is a crucial feature of evaluating an aptasensor performance. To investigate the selectivity of the designed aptasensor, other mycotoxins, including AFB1, AFM1 and ZEN, were tested. A comparison of the obtained results, shown in Fig. 5, indicates that the response signal to the FB1 was significantly higher than other mycotoxins. Moreover, it was found that by the addition of interfering substances such as AFB1, AFM1, and ZEN to the solution containing the target FB1, the response signal was close to that of the FB1. This demonstrated the outstanding selectivity of the prepared aptasensor toward FB1, which makes it suitable for use in practical applications.

### Reproducibility, repeatability and stability of the designed aptasensor

**Fig. 6:**
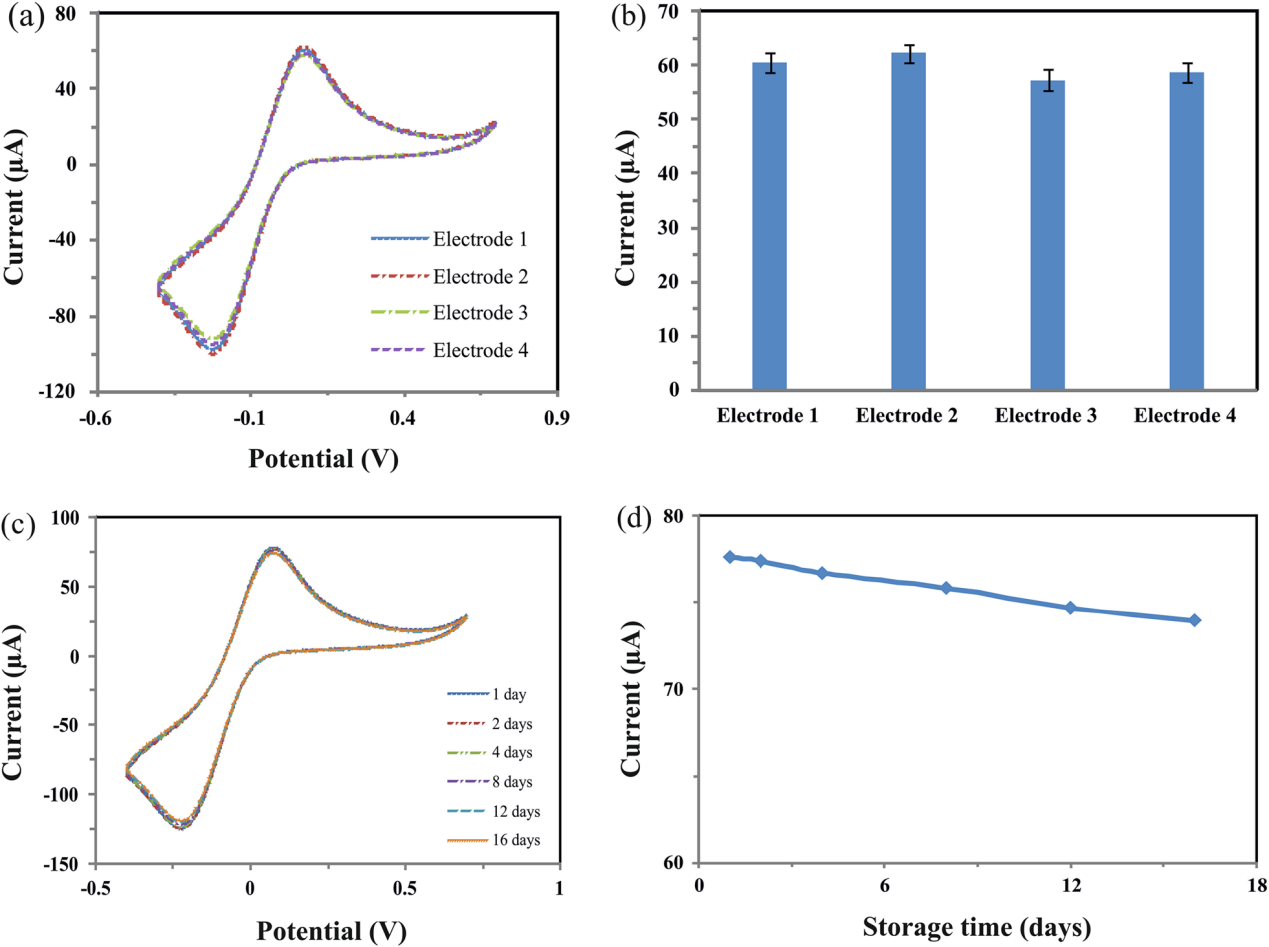
**a** Cyclic voltammograms and **b** current oxidation peaks related to reproducibility, **c** cyclic voltammograms and **d** current oxidation peaks of the ss-HSDNA/AuNPs/SPCE stored during 16 days

To evaluate the reproducibility and repeatability of the designed aptasensor, the response current to detect FB1 was measured six times at each of the four electrodes prepared independently in the same approach (Fig. 6a, b). The aptasensor exhibits good reproducibility and repeatability with a relative standard deviation (RSD) of 2.21% and 1.87%, respectively. As can be seen in Fig. 6c and d, the response signal reached 95% of its initial signal after 16 days of storage period at 4 °C, indicating the excellent long-term stability of the aptasensor.

### Maize sample analysis

The performance of the designed FB1apatsensor in practical applications was investigated using the recovery experiments on maize samples spiked with different FB1 concentrations (Table 2). Moreover, the obtained results were also checked by HPLC analysis. Based on Table 2, the recovery value of the designed aptasensor for maize samples was obtained 96–103%, which was consistent with HPLC results. The recovery ranges show that the CV aptasensor can be used to identifyFB1 in maize samples with adequate reliability.

**Table 2:** Comparison of electrochemical aptasensor assay with HPLC for detection FB1 in real sample

FB1 added (ng/mL)	Aptasensor		HPLC	
	FB1 founded (ng/mL)	Recovery %	FB1 founded (ng/mL)	Recovery %
10	10.3	103	9.8	98
50	48.1	96	53.1	106
100	102.2	102	103.4	103
200	197.3	99	204.7	102

## Conclusion

In the current research, a novel and competitive electrochemical aptasensor based on immobilization of ss-HSDNA on SPCE modified with AuNPs was developed for the determination of FB1 in maize samples. The cyclic voltammetry was used to monitor the variation of electron transfer occurring in the construction and utilization of the designed aptasensor. The obtained results can be briefly summarized as follows:

1. The simple and convenient aptasenor provided a linear range from 0.5 to 500 ng/mL and a detection limit of 0.14 ng/mL.
2. The designed aptasensor displayed excellent selectivity toward FB1 in the presence of other mycotoxins, including AFB1, AFM1, and ZEN.
3. The reproducibility, repeatability, and long-term stability of the proposed aptasensor toward FB1 detection were good.
4. 4-No significant differences in the precision and accuracy were found between the designed aptasensor and HPLC method.
5. These advantages demonstrated that the designed aptasensor could be recommended as a suitable screening tool for the detection of FB1 in food and feed.

## Data Availability

The datasets generated during and/or analyzed during the current study are available from the corresponding author on reasonable request.
